# The effectiveness of flower strips and hedgerows on pest control, pollination services and crop yield: a quantitative synthesis

**DOI:** 10.1111/ele.13576

**Published:** 2020-08-18

**Authors:** Matthias Albrecht, David Kleijn, Neal M. Williams, Matthias Tschumi, Brett R. Blaauw, Riccardo Bommarco, Alistair J. Campbell, Matteo Dainese, Francis A. Drummond, Martin H. Entling, Dominik Ganser, G. Arjen de Groot, Dave Goulson, Heather Grab, Hannah Hamilton, Felix Herzog, Rufus Isaacs, Katja Jacot, Philippe Jeanneret, Mattias Jonsson, Eva Knop, Claire Kremen, Douglas A. Landis, Gregory M. Loeb, Lorenzo Marini, Megan McKerchar, Lora Morandin, Sonja C. Pfister, Simon G. Potts, Maj Rundlöf, Hillary Sardiñas, Amber Sciligo, Carsten Thies, Teja Tscharntke, Eric Venturini, Eve Veromann, Ines M.G. Vollhardt, Felix Wäckers, Kimiora Ward, Duncan B. Westbury, Andrew Wilby, Megan Woltz, Steve Wratten, Louis Sutter

**Affiliations:** ^1^ Agroecology and Environment Agroscope Reckenholzstrasse 191 Zurich CH‐8046 Switzerland; ^2^ Plant Ecology and Nature Conservation Group Wageningen University Droevendaalsesteeg 3a Wageningen 6708PB The Netherlands; ^3^ Department of Entomology and Nematology and Graduate Group in Ecology University of California, Davis One Shields Ave Davis CA 95616 USA; ^4^ Department of Entomology University of Georgia Athens Georgia 30602 USA; ^5^ Department of Ecology Swedish University of Agricultural Sciences PO Box 7044 Uppsala 75007 Sweden; ^6^ Laboratório de Entomologia Embrapa Amazônia Oriental Belém Pará CEP 66095‐903 Brazil; ^7^ Institute for Alpine Environment Eurac Research Viale Druso 1 Bozen/Bolzano 39100 Italy; ^8^ School of Biology And Ecology University of Maine Orono ME 04469 USA; ^9^ iES Landau Institute for Environmental Sciences University of Koblenz‐Landau Fortstr. 7 Landau D‐76829 Germany; ^10^ University of Bern Institute of Ecology and Evolution Baltzerstrasse 6 Bern 3012 Switzerland; ^11^ Wageningen Environmental Research Wageningen University & Research P.O. Box 47 Wageningen 6700 AA The Netherlands; ^12^ School of Life Sciences University of Sussex Brighton BN1 9QG UK; ^13^ Department of Entomology Cornell University Geneva NY 14456 USA; ^14^ Department of Entomology and EEBB Program Michigan State University East Lansing MI 48824 USA; ^15^ Institute for Resources, Environment and Sustainability, & Department of Zoology University of British Columbia Vancouver V6T 1Z4 Canada; ^16^ Department of Entomology and Great Lakes Bioenergy Research Center Michigan State University East Lansing MI 48824 USA; ^17^ DAFNAE University of Padova viale dell’Università 16 Padova 35020 Italy; ^18^ School of Science & the Environment University of Worcester Worcester WR2 6AJ UK; ^19^ Pollinator Partnership 475 Sansome Street, 17th Floor San Francisco CA 94111 USA; ^20^ Centre for Agri‐Environmental Research School of Agriculture, Policy and Development Reading University Reading RG6 6AR UK; ^21^ Department of Biology Lund University Lund 223 62 Sweden; ^22^ Department of Environmental Science, Policy, and Management University of California 130 Mulford Hall Berkeley CA 94720 USA; ^23^ Agroecology Department of Crop Sciences University of Göttingen Göttingen Germany; ^24^ Wild Blueberry Commission of Maine 5784 York Complex, Suite 52 Orono Maine 04469 USA; ^25^ Estonian University of Life Sciences Kreutzwaldi 1 Tartu 51006 Estonia; ^26^ Lancaster Environnent Centre Lancaster University LA1 4YQ UK; ^27^ Bio‐Protection Research Centre Lincoln University Lincoln New Zealand

**Keywords:** Agroecology, agri‐environment schemes, bee pollinators, conservation biological control, ecological intensification, farmland biodiversity, floral enhancements, natural pest regulation, pollination reservoirs, sustainable agriculture, wildflower strips

## Abstract

Floral plantings are promoted to foster ecological intensification of agriculture through provisioning of ecosystem services. However, a comprehensive assessment of the effectiveness of different floral plantings, their characteristics and consequences for crop yield is lacking. Here we quantified the impacts of flower strips and hedgerows on pest control (18 studies) and pollination services (17 studies) in adjacent crops in North America, Europe and New Zealand. Flower strips, but not hedgerows, enhanced pest control services in adjacent fields by 16% on average. However, effects on crop pollination and yield were more variable. Our synthesis identifies several important drivers of variability in effectiveness of plantings: pollination services declined exponentially with distance from plantings, and perennial and older flower strips with higher flowering plant diversity enhanced pollination more effectively. These findings provide promising pathways to optimise floral plantings to more effectively contribute to ecosystem service delivery and ecological intensification of agriculture in the future.

## Graphical abstract

Our quantitative synthesis of the impacts of floral plantings on pest control, crop pollination and yield reveals that flower strips, but not hedgerows, enhanced pest control services in adjacent fields by 16% on average, while effects on crop pollination and yield were more variable. Our synthesis identifies several important drivers of this variability in effectiveness of plantings: pollination services declined exponentially with distance from plantings, and perennial and older flower strips with higher flowering plant diversity enhanced pollination more effectively, with important implications for the the design and implementation of these measures to effectively promote ecological intensification of agriculture in the future

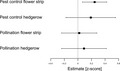

## Introduction

Meeting the increasing demands for agricultural products while minimising negative impacts on biodiversity and ecosystem health is among the greatest global challenges (Godfray *et al*., 2010). Intensive agricultural production and the simplification of agroecosystems threaten farmland biodiversity and associated ecosystem services worldwide (Foley *et al*., 2005; IPBES, 2016; IPBES, 2018). Concerns over loss of biodiversity and associated impairment of ecosystem services have helped strengthen the implementation of agri‐environmental schemes and other measures to mitigate such negative consequences (IPBES, 2016). Beyond restoration of farmland biodiversity in general, an implicit or explicit goal of such measures is to foster sustainable agricultural production through ecological intensification by harnessing biodiversity‐based ecosystem services, such as crop pollination and natural pest control services (Bommarco *et al*., 2013; Pywell *et al*., 2015; Kovács‐Hostyánszki *et al*., 2017). In intensively managed agroecosystems, the establishment of strips or other areas of flowering herbaceous plants, hereafter ‘flower strips’, and hedgerows are among the most commonly applied measures to achieve these goals (Scheper *et al*., 2015; Tschumi *et al*., 2015; Williams *et al*., 2015; Dainese *et al*., 2017; Kremen *et al*., 2019). For example, the establishment of flower strips or hedgerows is supported by the Common Agricultural Policy (CAP) in the European Union and by the Farm Bill (e.g. programs of the Natural Resources Conservation Service of the United States Department of Agriculture) in the United States (IPBES, 2016; Kovács‐Hostyánszki *et al*., 2017; Venturini *et al*., 2017a). Typically established along field edges, flower strips and hedgerows offer resources for pollinators and natural enemies of crop pests such as shelter, oviposition sites, overwintering opportunities and food resources (Tschumi *et al*., 2015; Holland *et al*., 2016; Kremen *et al*., 2019) and can locally increase their abundance and diversity (Haaland *et al*., 2011; Scheper *et al*., 2013; M’Gonigle *et al*., 2015; Williams *et al*., 2015; Tschumi *et al*., 2016; Sutter *et al*., 2017, 2018; Kremen *et al*., 2019). It is less well understood whether enhanced species diversity translates to *ex situ* provisioning of pollination, pest control and increased yield. The ‘exporter’ hypothesis (Morandin and Kremen, 2013; Kremen *et al*., 2019) predicts enhanced delivery of ecosystem services through functional spillover from floral plantings (*sensu* Blitzer *et al*., 2012; Albrecht *et al*., 2007; Morandin and Kremen, 2013; Pywell *et al*., 2015; Tschumi *et al*., 2015, 2016; Sutter *et al*., 2017). However, according the ‘concentrator’ hypothesis (Kremen *et al*., 2019; also referred to as the ‘aggregation’ hypothesis (Venturini *et al*., 2017a) or the ‘Circe principle’ (Lander *et al*., 2011)), resource‐rich floral plantings temporarily compete with flowering crops and concentrate pollinators and natural enemies from the surrounding agriculture into the floral plantings, potentially resulting in (transiently) reduced crop pollination and pest control services (Nicholson *et al*., 2019). This may explain why plantings fail to enhance crop pollination or pest control services, even if they successfully promote local pollinator or natural enemy abundance in restored habitats (e.g. Phillips and Gardiner, 2015; Tscharntke *et al*., 2016; Karp *et al*., 2018).

The lack of clarity about effects of flower plantings on ecosystem service provisioning and crop yield scattered in numerous case studies is a barrier to farmer adoption of such measures (Garbach and Long, 2017; Kleijn *et al*., 2019). A quantitative synthesis of such demonstrated broad evidence may assist farmers in making the decision to adopt these measures (Garbach and Long, 2017; Kleijn *et al*., 2019). Moreover, it is important to gain a general understanding of whether such effects are restricted to the area of the crop near to the adjacent planting (Ganser *et al*., 2018) or be detectable over larger distances (Tschumi *et al*., 2015). Such knowledge should be considered when designing schemes with optimal spatial arrangement of plantings across agricultural landscapes (Ricketts *et al*., 2008; Garibaldi *et al*., 2011), and to facilitate cost‐benefit assessments (Blaauw and Isaacs, 2014; Morandin *et al*., 2016; Dainese *et al*., 2017; Williams *et al*., 2019; Haan *et al*., 2020).

To improve the effectiveness of flower strip and hedgerow plantings in promoting crop pollination, natural pest control, and potentially crop production, we need to better understand what determines their failure or success. We hypothesise that at least three factors influence the effectiveness of floral plantings in enhancing crop pollination and pest control services: plant diversity, time since establishment and landscape context. First, theory predicts that higher plant species richness, and associated trait diversity, promotes diverse pollinator and natural enemy communities due to positive selection and complementarity effects across space and time (e.g. Campbell *et al*., 2012; Scheper *et al*., 2013; Sutter *et al*., 2017; M’Gonigle *et al*., 2017). However, the role of plant diversity driving effects of floral plantings on pollination and natural pest control services benefits to nearby crops is poorly understood. Second, time since the establishment of floral plantings is likely to play a key role for the local delivery of crop pollination and pest control services (Thies and Tscharntke, 1999). This is of particular relevance for sown flower strips that may range from short‐lived annual plantings to longer‐lived perennial plantings. Perennial plantings should offer better overwintering and nesting opportunities for pollinators and natural enemies (Ganser *et al*., 2019; Kremen *et al*., 2019) and may foster local population growth over time (e.g. Blaauw and Isaacs, 2014; Venturini *et al*., 2017b). Third, the effectiveness of floral plantings could depend on the agricultural landscape context. Highly simplified landscapes likely have depleted source populations of pollinators and natural enemies. In complex landscapes, however, the ecological contrast introduced by floral plantings may not be great enough to result in strong effects (Scheper *et al*., 2013). Strongest effects are therefore expected at intermediate landscape complexity (intermediate landscape complexity hypothesis; Tscharntke *et al*., 2005; Kleijn *et al*., 2011). Although support for this hypothesis has been found with respect to biodiversity restoration (e.g. Bátary *et al*., 2011; Scheper *et al*., 2013, 2015; but see e.g. Hoffmann *et al*., 2020), its validity for ecological intensification and the local delivery of crop pollination and pest control services has only just begun to be explored (Jonsson *et al*., 2015; Grab *et al*., 2018; Rundlöf *et al*., 2018).

Here we use data from 35 studies including 868 service‐site‐year combinations across 529 sites in North American, European and New Zealand agroecosystems to quantitatively assess the effectiveness of two of the most commonly implemented ecological intensification measures, flower strips and hedgerows, in promoting crop pollination, pest control services and crop production. Moreover, we aim to better understand the key factors driving failure or success of these measures to suggest improvement of their design and implementation. Specifically, we address: (1) the extent to which flower strips and hedgerows enhance pollination and pest control services in adjacent crops; (2) how service provisioning changes with distance from floral plantings; (3) the role of plant diversity and time since establishment of floral plantings in promoting pollination and pest control services; (4) whether simplification of the surrounding landscape modifies the responses; and (5) whether floral plantings enhance crop yield in adjacent fields.

Our synthesis reveals general positive effects of flower strips but not hedgerows on pest control services in adjacent crop fields. Effects on crop pollination, however, depended on flowering plant diversity and age since establishment, with more species‐rich and older plantings being more effective. However, no consistent impacts of flower strips on crop yield could be detected, highlighting the need for further optimisations of plantings as measures for ecological intensification.

## Materials and Methods

### Data collection

To identify data sets suitable to address our research questions, we performed a search in the ISI Web of Science and SCOPUS (records published until 31.12.2017 were considered). To minimise potential publication bias (i.e. the file drawer problem, Rosenthal 1979) and to maximise the number of relevant data sets we also searched for unpublished data by contacting potential data holders through researcher networks. Data sets had to meet the following requirements to be included in the analysis: (1) pollination and/or pest control services in crops were measured in both crop fields adjacent to floral plantings and control fields without planting; (2) the replication at the field level was ≥ six fields per study (three fields with plantings and three without; i.e. disqualifying small‐scaled plot treatment comparisons within fields). We contacted data holders fulfilling these requirements and requested primary data on plant species richness of plantings, time since establishment, landscape context and crop yield (see below) in addition to measured pollination and pest control services. Overall, we analysed data from 35 studies. We here define a study as a dataset collected by the same group of researchers for a particular crop species and ecosystem service (pest control or pollination) in a particular region during one or several sampling years. We collected 18 pest control service and 17 pollination service studies, representing a total of 868 service‐site‐year combinations across 529 sites (fields with or without adjacent floral planting; see Fig. S1 for a map showing the distribution of sites and Table S1 for detailed information about studies). In eight of these studies (122 sites) both crop pollination and pest control services were measured (Table S1).

### Pollination services, pest control services and crop yield

As different studies used different methods and measures to quantify pollination services, pest control services and crop yield, we standardised data prior to statistical analysis using *z*‐scores (e.g. Garibaldi *et al*., 2013; Dainese *et al*., 2019). The use of *z*‐scores has clear advantages compared with other transformations or standardisation approaches (such as the division by the absolute value of the maximum observed level of the measured response) because (1) average *z*‐scores follow a normal distribution, and (2) the variability present in the raw data is not constrained as in other indices that are bound between 0 and 1 (Garibaldi *et al*., 2013). Pollination services were measured as seed set (number of seeds per fruit), fruit set (proportion of flowers setting fruit), pollen deposition rate (number of pollen grains deposited on stigmas within a certain time period) and, in one study, flower visitation rate (number of visits per flower within a certain time period). If available, differences in pollination service measures of open‐pollinated flowers and flowers from which pollinators were excluded were analysed. Measures of pest control services were quantified as pest parasitism (proportion of parasitised pests), pest predation (proportion of predated pests), population growth (see below) or crop damage by pests or pest densities (see Table S2 for an overview of pollination and pest control service measures across studies). Whenever possible, the pest control index based on population growth proposed by Gardiner *et al*. (2009) was calculated and analysed (Table S2). Note that standardised values of pest density and crop damage were multiplied by −1 because lower values of these measures reflect an increased pest control service (e.g. Karp *et al*., 2018). Crop yield was only considered for the analysis if a direct measure of final crop yield was available. Too few studies assessed crop quality which was therefore not considered further. Yield was measured as crop mass or number of fruits produced per unit area. Due to a lack of studies measuring crop yield in fields with and without adjacent hedgerows, the analysis of crop yield focused on effects of flower strips. Crop yield measures were available from a total of 11 flower strip studies and 194 fields (see Tables S1 and S2 for a detailed description of study systems, crop yield measures and methods used across studies).

### Descriptors of floral plantings and landscape context

Flower strips are here defined as strips or other areas of planted wild native and/or non‐native flowering herbaceous plants. Hedgerows are defined as areas of linear shape planted with native and/or non‐native at least partly flowering woody plants and typically also herbaceous flowering plants. For hedgerows, information about the exact time since establishment and number of plant species was not available for most studies. The analyses of these drivers (question 3) therefore focus on flower strip effects on pollination and pest control services. Information on plant species richness was available in 12 out of 18 pest control studies and 10 out of 17 pollination studies. Whenever available, the species richness of flowering plants was used. Otherwise, for some flower strip studies, the number of sown, potentially flowering plant species (excluding grasses) was used. Time since establishment of flower strips, that is the time span between seeding or planting and data sampling, was available for all studies ranging from 3 to 122 months.

The proportional cover of arable crops was available and analysed as a proxy for landscape simplification (e.g. Tscharntke *et al*., 2005; Dainese *et al*., 2019) in 11 pest control and 12 pollination studies. Proportional cover of arable crops was calculated in circular sectors of 1 km radius around focal crops, or 750 m or 500 m radius (two studies for which data on a 1 km radius were not available; see Table S1; results remained qualitatively identical when only considering the 1 km radius datasets).

### Statistical analysis

We used a mixed effect‐modelling approach to address our research questions. In all models, study was included as a random intercept to account for the hierarchical structure of the data with field measures nested within study. To assess whether flower strips and hedgerows enhanced pollination and pest control services in adjacent crops (question 1) linear mixed‐effect models with planting (field with or without planting) were separately fitted for flower strips and hedgerows for the response variables pollination service and pest control service. To test how the effects on service provisioning change with distance (continuous variable; meters) from plantings (question 2) and with landscape simplification (question 4) these explanatory variables and their interactions with the fixed effects described earlier were included in the models. Exploratory analyses showed that neither distance nor landscape simplification effects differed between flower strips and hedgerows; that is no significant interactive effects of planting type with any of the tested fixed effects. We therefore pooled flower strip and hedgerow data in the final models, excluding planting type and its two or three‐way interactions as fixed effects. In addition to linear relationships we tested for an exponential decline of measured response variables from the border of the field by fitting log10(distance) in the linear mixed‐effect models described earlier. In this case, field nested within study was included as a random effect. To test the intermediate landscape complexity hypothesis, we tested for linear as well as hump‐shaped relationships between landscape context, and its interaction with local floral plantings by fitting landscape variables as a quadratic fixed predictor in the models described earlier (second degree polynomial functions). To present the ranges covered by the agricultural landscape gradients, we did not standardise measures of landscape simplification within studies (e.g. Martin *et al*., 2019). To examine how pollination and pest control service provisioning relates to flower strip plant diversity and time since establishment (question 3) plant species richness and log10(number of months since establishment) were included as fixed effects in models with study as a random effect. Using log(months since establishment) predicted the data better than establishment time as linear predictor. Plant species richness and time since establishment of flower strips were not correlated (*r* = 0.22). Only 10 studies measured services in several years since establishment (Table S1), and we included only data from the last sampling year. To assess how the presence of plantings affected the agronomic yield of adjacent crops (question 5), we fitted a linear mixed‐effect model with the same fixed and random structure as described for question 1, but with crop yield as the response variable. Statistical analyses for different models and response variables differed in sample sizes as not all studies measured crop yield in addition to pollination or pest control services (Tables 1, Table S1). In all models we initially included planting area as a co‐variate in an explorative analysis, but removed it in the final models, as it did not explain variation in any of the models and did not improve model fit (not shown).

**Table 1:** Summary of results of linear and generalised linear mixed‐effects models testing the effects of presence and type of floral plantings (flower strips and hedgerows) on crop pollination and natural pest control services, and how effects are influenced by in‐field distance, local planting characteristics and landscape context. Response variables, explanatory variables, estimates, numerator degrees of freedom and denominator degrees of freedom (Df), differences in log‐likelihood for chi‐squared tests (LRT) and *P* values (*P* < 0.05 in bold; *P* ≥ 0.05 < 0.10 in bold italic) are shown for each model. Note that effects of local drivers (i.e. flowering plant species richness and time since establishment) considered only crops adjacent to flower strips

Response variable	Explanatory variable	Estimate	Df	LRT	*P*‐value
Effects of plantings					
Natural pest control service	Flower strip	0.254	1,316	7.26	**0.007**
	Hedgerow	0.196	1,60	1.06	0.303
Crop pollination service	Flower strip	0.032	1,170	0.06	0.808
	Hedgerow	0.097	1,106	0.28	0.595
Distance effects					
Natural pest control service	Planting × log(distance)	−0.051	1,590.9	1.35	0.245
	Planting	0.199	1,590.4	5.92	**0.015**
	Log(distance)	−0.052	1,618.5	5.62	**0.018**
Crop pollination service	Planting × log(distance)	−0.082	1,445.3	5.73	**0.017**
	Planting	0.315	1,420.8	2.40	0.121
	Log(distance)	−0.014	1,453.3	2.64	0.104
Effects of local drivers (flower strips)					
Natural pest control service	Flowering plant species richness	−0.013	1,49.3	0.47	0.494
	Log(time since establishment)	0.104	1,16.1	1.32	0.251
Crop pollination service	Flowering plant species richness	0.036	1,49.8	3.39	***0.066***
	Log(time since establishment)	0.276	1,10.9	3.47	***0.062***
Effects of landscape context					
Natural pest control service	Planting × landscape simplification	−0.004	1,274.2	0.10	0.754
	Planting	0.171	1,286.2	1.28	0.257
	Landscape simplification	−0.007	1,181.9	1.81	0.179
Crop pollination service	Planting × landscape simplification	−0.003	1,278.9	0.91	0.340
	Planting	0.198	1,278.9	0.00	0.950
	Landscape simplification	−0.011	1,145.9	4.03	**0.045**

Effect sizes provided in the text and figures are model estimates of z‐transformed response variables. For statistical inference of fixed effects we used log‐likelihood ratio tests (LRT) recommended for testing significant effects of *a priori* selected parameters relevant to the hypotheses (Bolker *et al*., 2009). For all models, assumptions were checked according to the graphical validation procedures recommended by Zuur *et al*. (2009). All statistical analyses were performed in *R* version 3.5.2 (R Core Team, 2017) using the *R*‐package *lme4* (Bates *et al*., 2015).

## RESULTS

### Effects of floral plantings on pest control and pollination services

**Figure 1:**
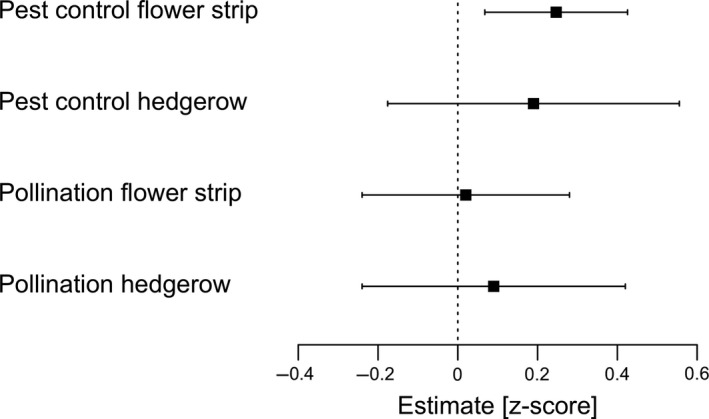
Forest plot showing effects of flower strips and hedgerows on pollination and pest control service provisioning in adjacent crops compared to control crops without adjacent floral plantings. Squares illustrate predicted mean effects (z‐score estimates), bars show 95% confidence intervals (CIs). On average, pest control services were enhanced by 16% (*z*‐score: 0.25) in fields with adjacent flower strip compared to control fields.

**Figure 2:**
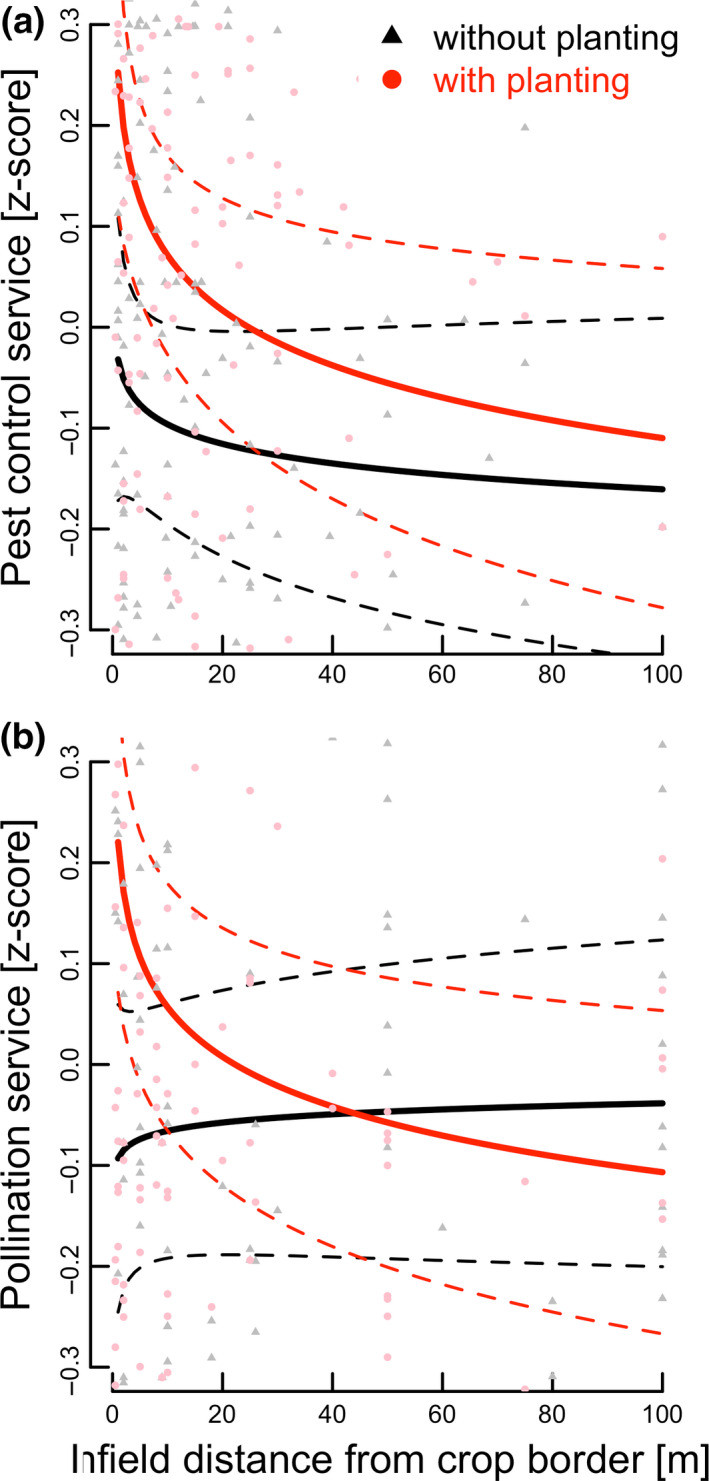
Predicted relationships between (a) mean natural pest control service and (b) mean crop pollination service (*z*‐scores (solid lines) ± 95% CI (dashed lines)) and in‐field distance to field border for field with (red lines; dots) or without adjacent floral planting (black lines, triangles).

The provisioning of pest control services in crop fields adjacent to flower strips was enhanced by 16% on average compared to fields without flower strips. On average, pest control services were also increased in crops adjacent to hedgerows, but effects were more variable and overall not statistically significant (Fig. 1; Table 1). Pest control services declined exponentially with distance from the field edge, but the slopes of the distance functions between fields with and without adjacent floral plantings did not differ (Fig. 2a; Table 1).

Crop pollination effects were more variable across studies and overall not significantly different between crops with or without adjacent floral planting across all studies and within‐field distances (Fig 1; Table 1). However, effects of distance to field edge differed for fields with floral plantings compared with control fields (significant interaction between presence of planting and distance from field border; Table 1). Pollination services were increased near floral plantings and decreased exponentially with increasing distance from plantings, while no such effect of distance to field edge was detected for control fields (Fig. 2b). The fitted distance curves for fields with or without floral plantings intersected at 43 m (Fig. 2b).

### The role of flowering plant diversity and time since establishment of flower strips

**Figure 3:**
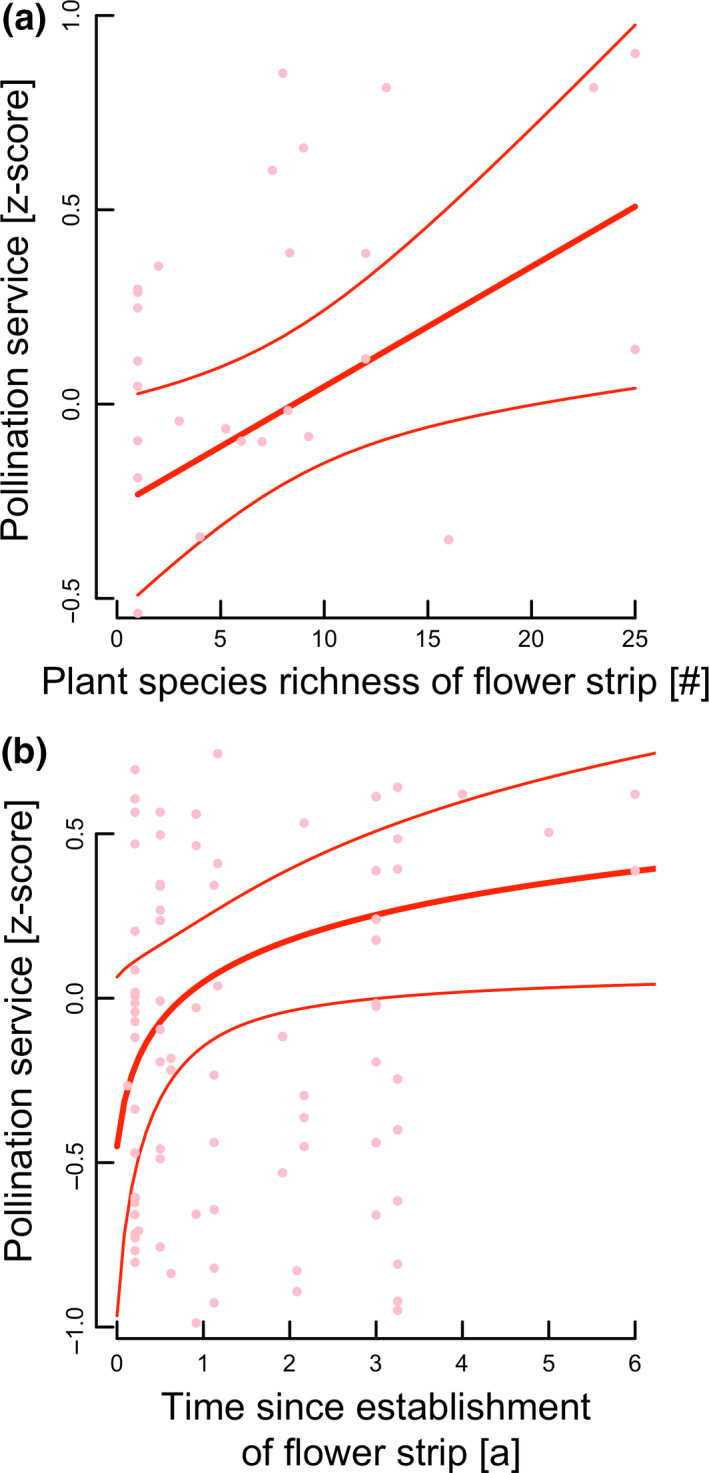
Predicted relationships between mean crop pollination service (*z*‐scores (fat solid lines) ± 95% CI (fine solid lines)) and (a) flowering plant species richness and (b) time since establishment of adjacent flower strips. Predicted relationship and results of an analysis without the points representing flower strips older than four years were qualitatively identical.

Crop pollination services, but not pest control services, tended to increase with flowering plant species richness of the adjacent flower strip (52% predicted increase in crop pollination from 1 to 25 plant species in adjacent flower strip; Fig. 3a; Table 1). Crop pollination services also tended to increase with time since establishment of the adjacent flower strip, but showed a positive saturating relationship (Fig. 3b; Table 1). Pollination services increased by 27% in 2 year old strips compared with the youngest plantings (roughly 3 months old), while the additional predicted increase from 2 to 4 years or older strips was approximately 5% on average (Fig. 3b; only few strips were older than four years, see Fig. 3b and explanations in figure caption). Pest control services in crops adjacent to flower strips did not increase with flower strip age (Table 1).

### Effects of landscape simplification

**Figure 4:**
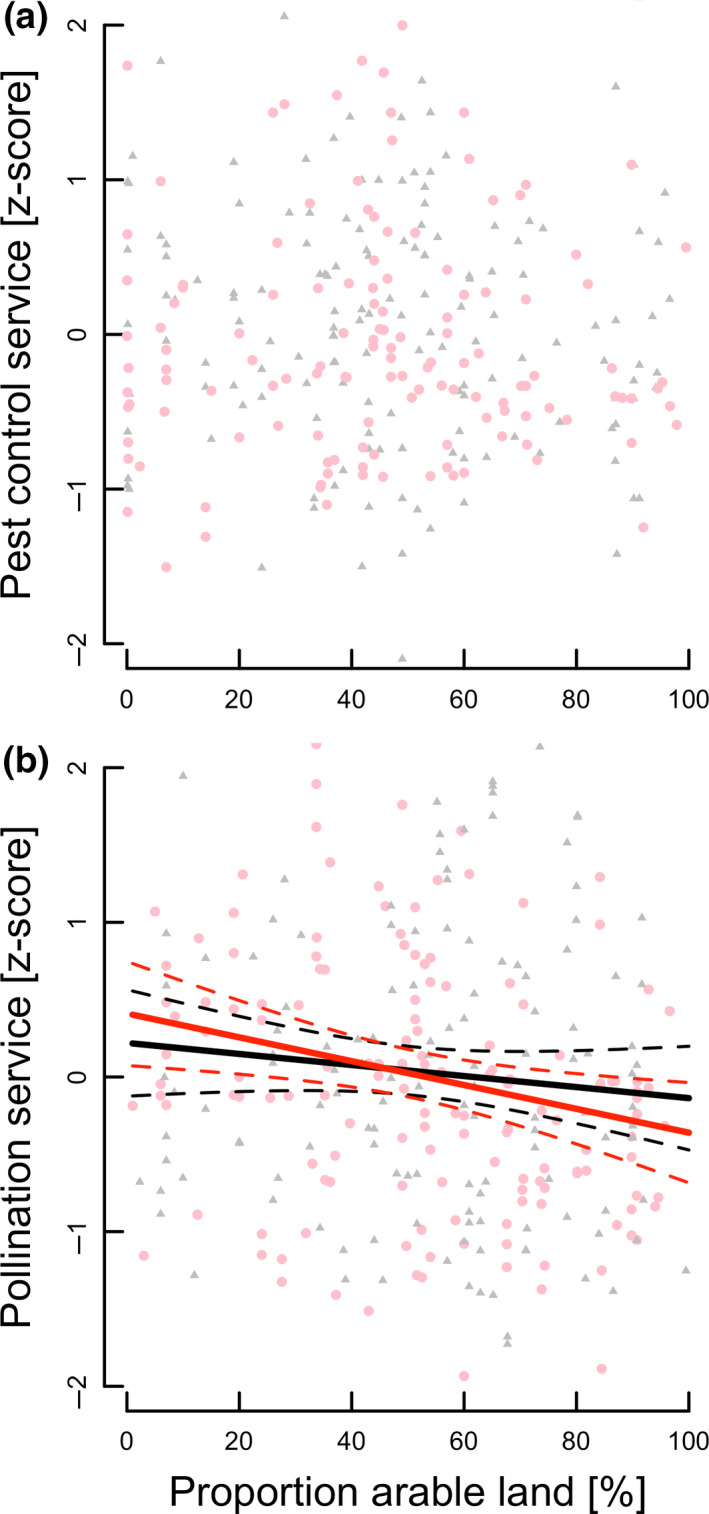
Predicted relationship between mean (a) pest control and (b) crop pollination service (*z*‐scores (solid lines) ± 95% CI (dashed lines)) and landscape simplification (percentage of arable crops in the landscape) in fields with adjacent floral planting (red line; red circles) or without planting (black line; black triangles). Pollination services, but not pest control services, declined with landscape simplification; the slight differences in slopes for pollination‐landscape simplification relationships of fields with or without adjacent plantings were statistically not significant.

The model testing for a linear relationship between service provision and landscape simplification and its interaction with local flower presence fitted the data better than a model testing for hump‐shaped relationships (Table S3). Pollination, but not pest control services, decreased linearly with landscape simplification (12% decrease from 50 to 100% crops in the surrounding landscape), irrespective of the presence of a floral planting (no significant floral planting × landscape simplification interaction; Fig 4; Table 1).

### Effects of flower strips on crop yield

**Figure 5:**
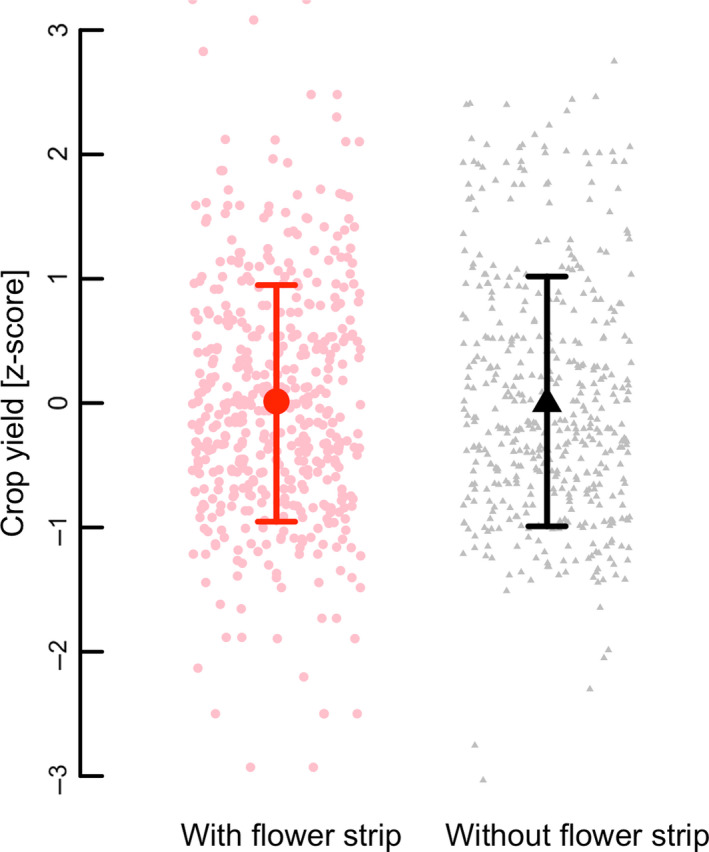
Mean predicted crop yield (*z*‐scores; ±95% CI) of fields with adjacent flower strips (red circles) and control fields without adjacent flower strip (black triangles). The data set includes a subset of 11 studies.

Overall, no significant effect of flower strips on yield in adjacent crops was detected (subset of 11 studies for which crop yield data was available; Fig. 5; Table S4). Furthermore, no effects of within‐field distance, plant species richness, time since establishment or landscape simplification, or their interactions with flower strip presence on yield, were detected (Table S4).

## DISCUSSION

Our quantitative synthesis demonstrates a generally positive effect of flower strips on pest control services but these effects did not consistently translate into higher yields. Although in most cases beneficial effects of plantings were also found for crop pollination services, effects on crop pollination and final crop yield were variable and overall not significant. The effect of wildflower strips on pollination services increased with age and species‐richness suggesting that the quality of such plantings plays a pivotal role in effective service provision. Moreover, crop pollination declined with increasing distance to floral plantings (hedgerows and flower strips). These results indicate that floral plantings have great potential to benefit ecosystem service provision, but to do so will need to be carefully tailored for functioning at specific spatial scales. Flower diversity and strip age are important drivers through which this can be achieved and they should be considered integrally before floral plantings can make a significant contribution to the ecological intensification of agricultural production.

We found positive effects of flower strips on ecosystem service provisioning in support of the ‘exporter’ hypothesis (*sensu* Morandin and Kremen, 2013; Kremen *et al*., 2019), although effects were generally variable and only significant for flower strips enhancing pest control services by 16% on average. This is an important finding as it provides general empirical evidence that flower strips can reduce crop pest pressures across various crops, landscape contexts and geographical regions. One explanation for the more consistent positive effects on pest control services of flower strips compared to hedgerows may be that in many of the studied flower strips the selection of flowering plants was tailored to the requirements of the target natural enemy taxa (Tschumi *et al*., 2015, 2016) while this was generally less the case in the studied hedgerow plantings.

Wildflower plantings have been heralded as one of the most effective measures to enhance the provision of ecosystem service to crops (Kleijn *et al*., 2019) with many studies showing positive effects on service provisioning (e.g. Blaauw and Isaacs, 2014; Tschumi *et al*., 2015, 2016; included in this quantitative synthesis). Our synthesis shows, however, that although general significant effects of flower strips were found for pest control service provisioning, effects of plantings on crop pollination services were highly variable. This highlights the need to better understand these conditions and drivers of success or failure of floral plantings to promote pollination services. Our synthesis identifies several drivers explaining this variability in delivered services and therefore offers pathways to enhance the effectiveness of these measures in the future.

First, the success of flower strips to promote crop pollination services increased with their age. The strongest increase was detected up to roughly three years since the planting date. Pollination services also appeared to continue to increase with establishment time beyond three years. This trend needs to be interpreted with caution as only three studies assessed four years old or older flower strips highlighting that scarcity of long‐term data on the effects of floral plantings on services provisioning and yield, which represents an important knowledge gap. We found no evidence that this increase in effectiveness with age is driven by floral abundance, as flower abundance did not increase with flower strip age. Case studies from Central and Northwestern Europe suggest that abundance and species richness of flowering herbaceous plants in sown flower strips on the highly fertilised soils in these agricultural regions often even decline with age after the second or third year as grasses take over (Steffan‐Dewenter and Tscharntke, 2001; Ganser *et al*., 2019). The observed positive effect of flower strip age is, however, in agreement with the expectation that the build‐up and restoration of local crop pollinator populations need time (Blaauw and Isaacs, 2014; Buhk *et al*., 2018; Kremen *et al*., 2018). It may also be explained by greater provision of nesting and overwintering opportunities in older floral plantings (Kremen *et al*., 2019) which are likely scarce in short‐lived annual flower strips that could even be ecological traps for overwintering arthropods (Ganser *et al*., 2019). In fact, Kremen and M’Gonigle (2015) found higher incidence of above‐ground cavity nesting bees compared to ground‐nesting bees with hedgerow maturation; Ganser *et al*. (2019) reported increased overwintering of arthropod predators and pollinators of perennial compared to annual flower strips.

Second, our findings reveal that higher species richness of flowering plants tends to enhance pollination service delivery in adjacent crops. This is an important finding as it indicates that restoring plant diversity can not only promote rare pollinator species and pollinator diversity (cf. Scheper *et al*., 2013; Kremen and M’Gonigle, 2015; Sutter *et al*., 2017; Kremen *et al*., 2018), but also crop pollination services. Flowering plant diversity likely promotes complementary floral resources for numerous pollinator taxa with different resource needs and continuity of floral resource availability throughout the season (Schellhorn *et al*., 2015; M’Gonigle *et al*., 2017). The identification of species or traits contributing particularly strongly to such effects is a promising area of research (Lundin *et al*., 2019). Moreover, appropriate management, such as reducing the frequency of hedgerow cutting, is important to ensuring high availability and diversity of floral resources (Staley *et al*., 2012). Our synthesis reveals that floral plantings enhance pollination services, but only in the part of adjacent crops near to plantings, declining exponentially with distance to plantings (Fig. 2). The exponential decline function predicts pollination service provisioning of less than 50% at 10 m and slightly more than 20% at 20 m compared to the level of service provisioning directly adjacent to plantings, partially explaining the overall non‐significant benefits when considering all measured distances across the entire field (Fig. 2). This may also explain part of the high variability observed across studies and reconcile some of the contrasting findings with respect to pollination service provisioning in studies measuring services relatively near plantings (e.g. up to 15 m; Blaauw and Isaacs (2014), or up to larger distances, e.g. up to 200 m; Morandin and Kremen (2013); Sardiñas *et al*, (2016)). We found no indication that the degree of the dependency of a crop on insect pollination significantly contributed the observed variability in effects of plantings on crop pollination services or yield (Table S5).

Consistent with previous studies (e.g. Dainese *et al*., 2019), landscape simplification was associated with decreased pollination services, irrespective of the presence of floral plantings. In contrast, no such effects were detected for pest control services, in agreement with recent studies (Karp *et al*., 2018; Dainese *et al*., 2019; but see Veres *et al*., 2013; Rusch *et al*., 2016; Martin *et al*., 2019). The effect of adding a flower strip or hedgerow was, however, independent of landscape context. Although individual case studies (Jonsson *et al*., 2015; Grab *et al*., 2018; included in this synthesis) found support for the intermediate landscape hypothesis, enhanced ecosystem services associated with floral plantings were not generally limited to moderately complex landscape contexts, which should encourage farmers to adopt these measures irrespective of the type of landscape in which they are farming.

Crop yield is affected by a complex interplay of a multitude of agricultural management practices such as fertilisation, level of pesticide use, pest pressures, soil cultivation and other factors such as local soil and climatic conditions (e.g. Bartomeus *et al*., 2015; Gagic *et al*., 2017), which can potentially mask benefits from improved natural pest regulation or pollination services (Sutter *et al*., 2018). Positive effects of floral plantings have been shown by some case studies included in this synthesis (e.g. Tschumi *et al*., 2016; see also Pywell *et al*., 2015), although sometimes only several years after the establishment of plantings (Blaauw and Isaacs, 2014; Morandin *et al*., 2016; Venturini *et al*., 2017b), but we did not detect consistent effects on crop yield associated with adjacent floral plantings. The identified drivers of the effectiveness of floral plantings to enhance crop pollination services, such as age and flowering plant diversity, could provide promising pathways towards optimising plantings as measures contributing to ecological intensification. Future optimisations should also consider the potential for synergistic interactions of enhanced pollination and pest control services by ‘multi‐service’ designs of plantings (Sutter and Albrecht, 2016; Morandin *et al*., 2016), temporal dynamics (Blaauw and Isaacs, 2014; M’Gonigle *et al*., 2015), optimised ratios of floral planting (contributing to ecosystem service supply) to crop area (affecting service demand; Kremen *et al*., 2019; Williams *et al*., 2019), and the distance‐dependency of services quantified by this synthesis. However, floral plantings are also established for other goals than yield increase. From an environmental and health perspective, maintaining crop yields through a replacement of insecticides by enhanced natural pest control services, should be considered as a great achievement (e.g. Tschumi *et al*., 2015). Moreover, floral plantings, of sufficient ecological quality, for example in terms of native plant species diversity, contribute also to further ecosystem services, especially biodiversity conservation (e.g. Haaland *et al*., 2011; Scheper *et al*., 2013); but farmers are often reluctant to adopt such measures due to concerns of negative effects on crop yield, for example due to spillover of pests. Our findings of similar crop yield in fields with and without plantings can dispel such concerns.

### Conclusions and implications

Our synthesis demonstrates enhanced natural pest control services to crops adjacent flower strips plantings, across a broad suite of regions, cropping systems and types of flower strips studied. However, it also reveals inconsistent and highly variable effects of flower strips and hedgerows on crop pollination services and yield. This highlights a strong need to identify the key factors driving this variability and the effectiveness of different types of floral plantings in contributing to ecosystem service delivery. Informed by such improved understanding, the design, implementation and management of floral plantings can increase their effectiveness as measures for ecological intensification. This synthesis identifies several promising pathways towards more effective floral plantings for the provision of ecosystem services and ecological intensification: the modelled exponential distance‐decay function of pollination service provisioning by floral plantings into crop field helps to predict service provision in crop fields; together with the lack of a strong planting area effect, our findings suggest that a dense spatial network of relatively small plantings will be more effective than a few large ones to optimise pollination service provisioning. Moreover, it identifies important drivers of the effectiveness of floral plantings for delivery of crop pollination services: flowering plant diversity and age. Based on these findings we strongly encourage the establishment, adequate management and restoration of existing perennial floral plantings that ensure the availability of high floral diversity across several years as promising pathways towards optimised measures for ecological intensification.

## Authors’ contributions

MA and LS designed the study. MA, DK, MT, BRB, RB, AJC, MD, FD, MHE, DG, ADG, DG, HG, HH, FH, RI, KJ, PJ, MJ, EK, CK, DAL, GML, LM, MMK, LM, SCP, SGP, MR, HS, AS, CT, TT, EV, EV, IMGV, AW, DBW, FW, KW, NMW, MW, SW and LS contributed data. MA compiled the dataset. LS and MA analysed the data. MA, LS, DK, MG, SGP and MR interpreted results. MA wrote the paper and all authors contributed to revision

## Supporting information

Fig S1

Table S1

Table S2

Table S3

Table S4

Table S5

## Data Availability

Data available from the Dryad Digital Repository: https://doi.org/10.5061/dryad.ns1rn8pq2.
