# Supplementary material for: The effectiveness of flower strips and hedgerows on pest control, pollination services and crop yield: a quantitative synthesis
Source: Ecol Lett. 2020 Aug 18;23(10):1488–98. doi: 10.1111/ele.13576 (PMC7540530; doi:10.1111/ele.13576)

*Supporting information* to Albrecht *et al.*: **The effectiveness of flower strips and hedgerows on pest control, pollination services and crop yield: a quantitative synthesis**

**Supporting Figure S1.** Maps of the geographic distribution of study locations: **a)** global overview of study regions in Europe, North America and New Zealand; maps of the geographic distribution of the study locations across the two global regions in which most studies were conducted: **b)** Europe and **c)** North America. Point size reflects the number of sites per study.

1. **Geographic distribution of study locations worldwide**


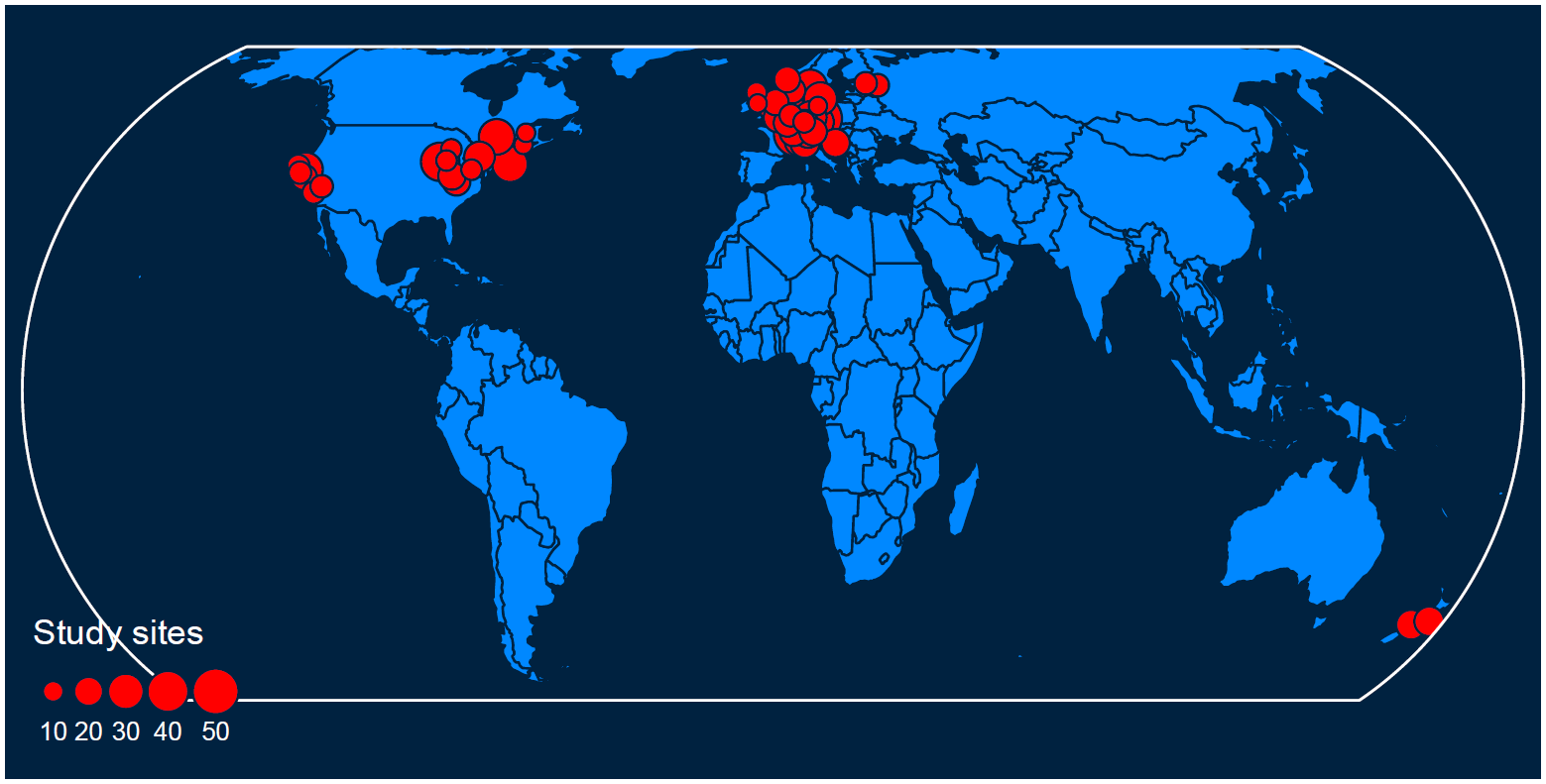


1.
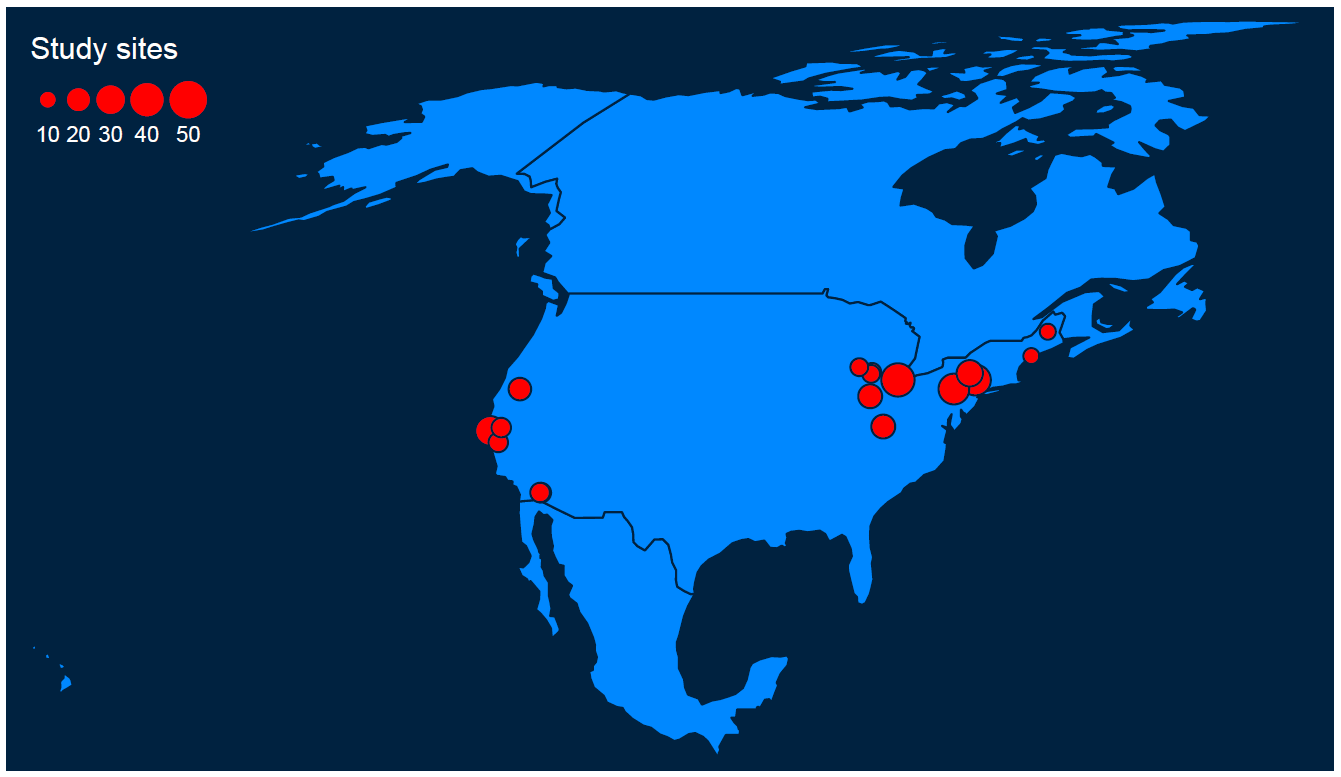
**Study locations in Europe c) Study locations in North America**


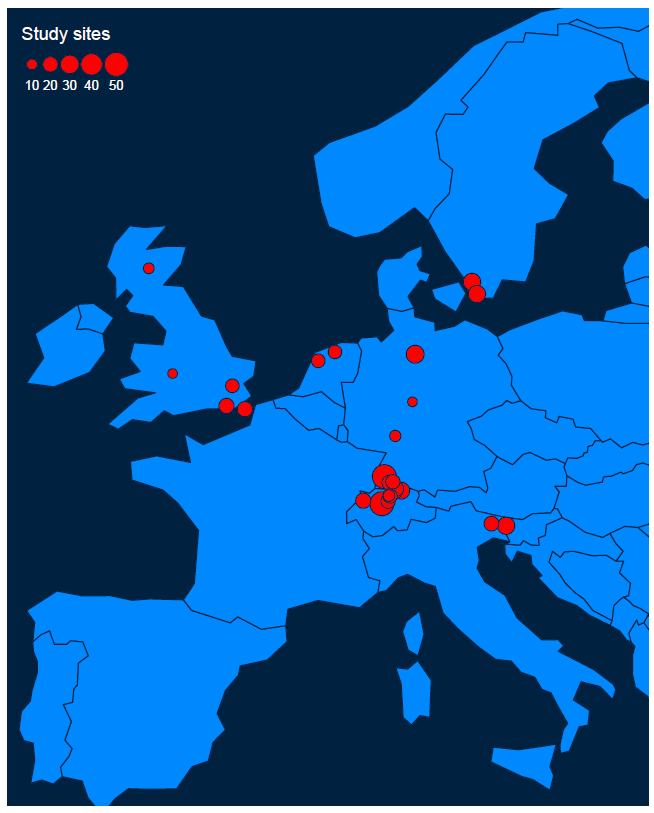

Supplement: Supplementary file 1 — Fig S1 [file ELE-23-1488-s005.docx]
