# Supplementary material for: The effectiveness of flower strips and hedgerows on pest control, pollination services and crop yield: a quantitative synthesis
Source: Ecol Lett. 2020 Aug 18;23(10):1488–98. doi: 10.1111/ele.13576 (PMC7540530; doi:10.1111/ele.13576)
Supplement: Supplementary file 2 — Table S1 [file ELE-23-1488-s004.docx]

*Supporting information* to Albrecht *et al.*: **The effectiveness of flower strips and hedgerows on pest control, pollination services and crop yield: a quantitative synthesis**

**Supporting Table S1.** Overview of studies considered in analyses. ‘Radius’ refers to the radius around focal fields in which landscape variables were assessed (see Material and methods section for detailed description of variables analysed).

|  |  |  |  |  |  |  |  |  |  |
| --- | --- | --- | --- | --- | --- | --- | --- | --- | --- |
| **Study ID** | **Type of  response** | **Planting type** | **Study region,  country** | **Crop species** | **Study year(s)** | **Sites** | **Radius (m)** | **Publication** |  |
|  |  |  |  |  |  |  |  |  |  |
| Blaa01 | pest control | flower strip | Michigan, USA | highbush blueberry  (*Vaccinium corymbosum*) | 2011-2012 | 10 | 1000 | Blaauw & Isaacs 2015 |  |
| Conn01 | pest control | flower strip | Finger Lakes, New York, USA | strawberry  (*Fragaria ananassa*) | 2014-2015 | 22 | 1000 | Grab *et al.* 2018 |  |
| Dain01 | pest control | flower strip | Veneto, Italy | winter wheat  (*Triticum aestivum*) | 2014 | 26 | 1000 | Dainese *et al.* 2017 |  |
| Entl01 | pest control | flower strip | Central Plateau, Switzerland | winter wheat  (*Triticum aestivum*) | 2005 | 20 | 1000 | unpublished |  |
| Gard01 | pest control | flower strip | Ohio, USA | pumpkin (*Cucurbita pepo*) | 2011-2012 | 18 | 1000 | Phillips & Gardiner 2016 |  |
| Groo01 | pest control | flower strip | Flevoland, The Netherlands | winter wheat  (*Triticum aestivum*) | 2014 | 16 | 1000 | unpublished |  |
| Jaco01 | pest control | flower strip | Central plateau, Switzerland | winter wheat  (*Triticum aestivum*) | 2016 | 50 | NA | unpublished |  |
| Jons01 | pest control | flower strip | Canterbury region, New Zealand | kale (*Brassica oleracea*) | 2008 | 21 | 1000 | Jonsson *et al.* 2015 |  |
| Mcke01 | pest control | flower strip | Kent, UK | apple (*Malus domestica*) | 2015 | 16 | 1000 | unpublished |  |
| Mora01 | pest control | hedgerow | Yolo County, California, USA | tomato (*Solanum lycopersicum*) | 2009-2010 | 12 | 1000 | Morandin *et al.* 2014 |  |
| Sche01 | pest control | flower strip | Lower Saxony, Germany | winter wheat  (*Triticum aestivum*) | 2005-2007 | 28 | 1000 | Scheid *et al.* 2011 |  |
| Sutt01 | pest control | hedgerow,  flower strip | Eastern Plateau, Switzerland | oilseed rape (*Brassica napus*) | 2014 | 18 | 1000 | Sutter *et al.* 2018 |  |
| Thie01 | pest control | flower strip | Lower Saxony, Germany | oilseed rape (*Brassica napus*) | 1995 | 8 | NA | Thies & Tscharntke 1999 |  |
| Tsch01 | pest control | flower strip | Eastern Plateau, Switzerland | winter wheat (*Triticum aestivum*) | 2012 | 25 | 1000 | Tschumi *et al.* 2015 |  |
| Tsch02 | pest control | flower strip | Eastern Plateau, Switzerland | potato (*Solanum tuberosum*) | 2013 | 18 | NA | Tschumi *et al.* 2016a |  |
| Tsch03 | pest control | flower strip | Eastern Plateau, Switzerland | winter wheat (*Triticum aestivum*) | 2014 | 20 | NA | Tschumi *et al.* 2016b |  |
| Vero01 | pest control | hedgerow | Tartu County, Estonia | oilseed rape (*Brassica napus*) | 2014 | 12 | 1000 | Kovács *et al.* 2019 |  |
| Wolt01 | pest control | flower strip | Michigan, USA | soybean (*Glycine max*) | 2008-2009 | 35 | 2000 | Woltz *et al.* 2012 |  |
| Blaa01 | pollination | flower strip | Michigan, USA | highbush blueberry  (*Vaccinium corymbosum*) | 2009-2012 | 10 | 1000 | Blaauw & Isaacs 2014 |  |
| Camp01 | pollination | flower strip | Herefordshire, UK | apple  (*Malus domestica*) | 2013 | 8 | NA | Campbell *et al.* 2017a,b |  |
| Conn01 | pollination | flower strip | Finger Lakes, New York, USA | strawberry  (*Fragaria ananassa*) | 2014-2015 | 30 | 1000 | Grab *et al.* 2018 |  |
| Dain01 | pollination | flower strip | Veneto, Italy | oilseed rape (*Brassica napus*) | 2014 | 19 | 1000 | Dainese *et al.* 2017 |  |
| Felt01 | pollination | flower strip | Meigle, Scotland | strawberry  (*Fragaria ananassa*) | 2013 | 10 | NA | Feltham *et al.* 2015 |  |
| Gans01 | pollination | flower strip | Eastern Plateau, Switzerland | strawberry  (*Fragaria ananassa*) | 2016-2017 | 18 | 1000 | Ganser *et al.* 2018 |  |
| Gard01 | pollination | flower strip | Ohio, USA | pumpkin (*Cucurbita pepo*) | 2011-2012 | 18 | 1000 | Phillips & Gardiner 2015 |  |
| Mcke01 | pollination | flower strip | Kent, UK | apple (*Malus domestica*) | 2013-2015 | 20 | 1000 | unpublished |  |
| Mora01 | pollination | hedgerow | Yolo County, California, USA | oilseed rape (*Brassica napus*) | 2010-2011 | 12 | 1000 | Morandin *et al.* 2016 |  |
| Pfis01 | pollination | hedgerow | Rhineland-Palatinate Germany | pumpkin (*Cucurbita maxima*) | 2014 | 11 | 1000 | Pfister *et al.* 2017 |  |
| Rund01 | pollination | flower strip | Skåne, Sweden | red clover (*Trifolium pratense*) | 2010 | 26 | 1000 | Rundlöf *et al.* 2018 |  |
| Sard01 | pollination | hedgerow | Yolo County, California, USA | sunflower (*Helianthus annuus*) | 2011-2013 | 28 | 1000 | Sardiñas & Kremen 2015 |  |
| Scil01 | pollination | hedgerow, flower strip | Central Coast, California, USA | strawberry (*Fragaria ananassa*) | 2012 | 16 | 1000 | unpublished |  |
| Sutt01 | pollination | hedgerow, flower strip | Eastern Plateau, Switzerland | oilseed rape (*Brassica napus*) | 2014 | 18 | 1000 | Sutter *et al.* 2018 |  |
| Vent01 | pollination | flower strip | Sargentville, Maine, USA | lowbush blueberry (*Vaccinium angustifolium*) | 2012-2013 | 8 | 1000 | Venturini *et al.* 2017 |  |
| Vero01 | pollination | hedgerow | Tartu County, Estonia | oilseed rape (*Brassica napus*) | 2014 | 12 | 1000 | unpublished |  |
| Will01 | pollination | flower strip | California, USA | watermelon (*Citrullus lanatus*) | 2011-2012 | 12 | NA | unpublished |  |
| Groo01 | yield | flowerstrip | Flevoland, The Netherlands | winter wheat  (*Triticum aestivum*) | 2014 | 16 | 1000 | unpublished |  |
| Jaco01 | yield | flowerstrip | Central Plateau, Switzerland | winter wheat  (*Triticum aestivum*) | 2016 | 50 | NA | unpublished |  |
| Jons01 | yield | flowerstrip | Canterbury region, New Zealand | kale (*Brassica oleracea*) | 2008 | 21 | 500 | Jonsson *et al.* 2015 |  |
| Sutt01 | yield | flowerstrip | Eastern Plateau, Switzerland | oilseed rape (*Brassica napus*) | 2014 | 12 | 1000 | Sutter *et al.* 2018 |  |
| Tsch03 | yield | flowerstrip | Eastern Plateau, Switzerland | winter wheat (*Triticum aestivum*) | 2014 | 20 | NA | Tschumi *et al.* 2016b |  |
| Blaa01 | yield | flowerstrip | Michigan, USA | highbush blueberry  (*Vaccinium corymbosum*) | 2009-2012 | 10 | 1000 | Blaauw & Isaacs 2014 |  |
| Conn01 | yield | flowerstrip | Finger Lakes, New York, USA | strawberry  (*Fragaria ananassa*) | 2014-2015 | 30 | 1000 | Grab *et al.* 2018 |  |
| Mcke01 | yield | flowerstrip | Kent, UK | apple (*Malus domestica*) | 2013-2015 | 20 | 1000 | unpublished |  |
| Rund01 | yield | flowerstrip | Skåne, Sweden | red clover (*Trifolium pratense*) | 2010 | 26 | 1000 | Rundlöf *et al.* 2018 |  |
| Vent01 | yield | flowerstrip | Sargentville, Maine, USA | lowbush blueberry (*Vaccinium angustifolium*) | 2012-2013 | 8 | 1000 | Venturini *et al.* 2017 |  |
| Will01 | yield | flowerstrip | California, USA | watermelon (*Citrullus lanatus*) | 2011-2012 | 12 | NA | unpublished |  |
|  |  |  |  |  |  |  |  |  |  |

**References**

Blaauw, B.R. & Isaacs, R. (2014). Flower plantings increase wild bee abundance and the pollination services provided to a pollination‐dependent crop. *J. Appl. Ecol.*, *51*, 890-898.

Blaauw, B R., & Isaacs, R. (2015). Wildflower plantings enhance the abundance of natural enemies and their services in adjacent blueberry fields. *Biol. Control*, *91*, 94-103.

Campbell, A., Wilby, A., Sutton, P. & Wäckers, F. (2017a). Getting more power from your flowers: Multi-functional flower strips enhance pollinators and pest control agents in apple orchards. *Insects*, *8*, 101.

Campbell, A. J., Wilby, A., Sutton, P. & Wäckers, F. L. (2017b). Do sown flower strips boost wild pollinator abundance and pollination services in a spring-flowering crop? A case study from UK cider apple orchards. *Agric. Ecosyst. Environ.*, *239*, 20-29.

Dainese, M., Montecchiari, S., Sitzia, T., Sigura, M., & Marini, L. (2017). High cover of hedgerows in the landscape supports multiple ecosystem services in Mediterranean cereal fields. *J. Appl. Ecol.*, *54*, 380-388.

Feltham, H., Park, K., Minderman, J., & Goulson, D. (2015). Experimental evidence that wildflower strips increase pollinator visits to crops. *Ecol. Evol.*, *5*, 3523-3530.

Ganser, D., Mayr, B., Albrecht, M., & Knop, E. (2018). Wildflower strips enhance pollination in adjacent strawberry crops at the small scale. *Ecol. Evol.*, *8*, 11775-11784.

Grab, H., Poveda, K., Danforth, B., & Loeb, G. (2018). Landscape context shifts the balance of costs and benefits from wildflower borders on multiple ecosystem services. *Proc. R. Soc. B*, *285*, 20181102.

Jonsson, M., Straub, C. S., Didham, R. K., Buckley, H. L., Case, B. S., Hale, R. J., ... & Wratten, S. D. (2015). Experimental evidence that the effectiveness of conservation biological control depends on landscape complexity. *J. Appl. Ecol.*, *52*, 1274-1282.

Kovács, G., Kaasik, R., Lof, M. E., van der Werf, W., Kaart, T., Holland, J. M., Luik, A. & Veromann, E. (2019). Effects of land use on infestation and parasitism rates of cabbage seed weevil in oilseed rape. *Pest Manag. Sci.*, *75*, 658-666.

Morandin, L.A., Long, R.F., & Kremen, C. (2014). Hedgerows enhance beneficial insects on adjacent tomato fields in an intensive agricultural landscape. *Agric. Ecosyst. Environ.*, *189*, 164-170.

Morandin, L. A., Long, R. F., & Kremen, C. (2016). Pest control and pollination cost–benefit analysis of hedgerow restoration in a simplified agricultural landscape. *J. Econ. Entomol.*, *109*, 1020-1027.

Phillips, B. W., & Gardiner, M. M. (2015). Use of video surveillance to measure the influences of habitat management and landscape composition on pollinator visitation and pollen deposition in pumpkin (*Cucurbita pepo*) agroecosystems. *PeerJ*, *3*, e1342.

Phillips, B. W., & Gardiner, M. M. (2016). Does local habitat management or large-scale landscape composition alter the biocontrol services provided to pumpkin agroecosystems?. *Biol. Control*, *92*, 181-194.

Pfister, S. C., Eckerter, P. W., Schirmel, J., Cresswell, J. E., & Entling, M. H. (2017). Sensitivity of commercial pumpkin yield to potential decline among different groups of pollinating bees. *R. Soc. Open Sci.*, *4*(5), 170102.

Rundlöf, M., Lundin, O., & Bommarco, R. (2018). Annual flower strips support pollinators and potentially enhance red clover seed yield. *Ecol. Evol.*, *8*, 7974-7985.

Sardiñas, H. S., & Kremen, C. (2015). Pollination services from field-scale agricultural diversification may be context-dependent. *Agric. Ecosyst. Environ.*, *207*, 17-25.

Scheid, B. E., Thies, C., & Tscharntke, T. (2011). Enhancing rape pollen beetle parasitism within sown flower fields along a landscape complexity gradient. *Agric. Forest Entomol.*, *13*, 173-179.

Sutter, L., Albrecht, M., & Jeanneret, P. (2018). Landscape greening and local creation of wildflower strips and hedgerows promote multiple ecosystem services. *J. Appl. Ecol.*, *55*, 612-620.

Thies, C., & Tscharntke, T. (1999). Landscape structure and biological control in agroecosystems. *Science*, *285*(5429), 893-895.

Tschumi, M., Albrecht, M., Entling, M. H., & Jacot, K. (2015). High effectiveness of tailored flower strips in reducing pests and crop plant damage. *Proc. R. Soc. B*, *282*, 20151369.

Tschumi, M., Albrecht, M., Collatz, J., Dubsky, V., Entling, M. H., Najar‐Rodriguez, A. J., & Jacot, K. (2016a). Tailored flower strips promote natural enemy biodiversity and pest control in potato crops. *J. Appl. Ecol.*, *53*, 1169-1176.

Tschumi, M., Albrecht, M., Bärtschi, C., Collatz, J., Entling, M. H., & Jacot, K. (2016b). Perennial, species-rich wildflower strips enhance pest control and crop yield. *Agric. Ecosyst. Environ.*, *220*, 97-103.

Venturini, E.M., Drummond, F.A., Hoshide, A.K., Dibble, A.C., & Stack, L.B. (2017). Pollination reservoirs in lowbush blueberry (Ericales: Ericaceae). *J. Econ. Entomol.*, *110*, 333-346.

Woltz, MJ, R Isaacs, DA Landis. 2012. Landscape structure and habitat management differentially influence insect natural enemies in an agricultural landscape. *Agric. Ecosyst. Environ.*, 152, 40-49.
