# Supplementary material for: The effectiveness of flower strips and hedgerows on pest control, pollination services and crop yield: a quantitative synthesis
Source: Ecol Lett. 2020 Aug 18;23(10):1488–98. doi: 10.1111/ele.13576 (PMC7540530; doi:10.1111/ele.13576)
Supplement: Supplementary file 3 — Table S2 [file ELE-23-1488-s003.docx]

*Supporting information* to Albrecht *et al.*: **The effectiveness of flower strips and hedgerows on pest control, pollination services and crop yield: a quantitative synthesis**

**Supporting Table S2.** Description of measurements of pest control and pollination services and crop yield performed in each study used in the analyses. See references for more detailed description of sampling methods.

**References**

Blaauw, B.R. & Isaacs, R. (2014). Flower plantings increase wild bee abundance and the pollination services provided to a pollination‐dependent crop. *J. Appl. Ecol.*, *51*, 890-898.

Blaauw, B R., & Isaacs, R. (2015). Wildflower plantings enhance the abundance of natural enemies and their services in adjacent blueberry fields. *Biol. Control*, *91*, 94-103.

Campbell, A., Wilby, A., Sutton, P. & Wäckers, F. (2017a). Getting more power from your flowers: Multi-functional flower strips enhance pollinators and pest control agents in apple orchards. *Insects*, *8*, 101.

Campbell, A. J., Wilby, A., Sutton, P. & Wäckers, F. L. (2017b). Do sown flower strips boost wild pollinator abundance and pollination services in a spring-flowering crop? A case study from UK cider apple orchards. *Agric. Ecosyst. Environ.*, *239*, 20-29.

Dainese, M., Montecchiari, S., Sitzia, T., Sigura, M., & Marini, L. (2017). High cover of hedgerows in the landscape supports multiple ecosystem services in Mediterranean cereal fields. *J. Appl. Ecol.*, *54*, 380-388.

Feltham, H., Park, K., Minderman, J., & Goulson, D. (2015). Experimental evidence that wildflower strips increase pollinator visits to crops. *Ecol. Evol.*, *5*, 3523-3530.

Ganser, D., Mayr, B., Albrecht, M., & Knop, E. (2018). Wildflower strips enhance pollination in adjacent strawberry crops at the small scale. *Ecol. Evol.*, *8*, 11775-11784.

Grab, H., Poveda, K., Danforth, B., & Loeb, G. (2018). Landscape context shifts the balance of costs and benefits from wildflower borders on multiple ecosystem services. *Proc. R. Soc. B*, *285*, 20181102.

Jonsson, M., Straub, C. S., Didham, R. K., Buckley, H. L., Case, B. S., Hale, R. J., ... & Wratten, S. D. (2015). Experimental evidence that the effectiveness of conservation biological control depends on landscape complexity. *J. Appl. Ecol.*, *52*, 1274-1282.

Kovács, G., Kaasik, R., Lof, M. E., van der Werf, W., Kaart, T., Holland, J. M., Luik, A. & Veromann, E. (2019). Effects of land use on infestation and parasitism rates of cabbage seed weevil in oilseed rape. *Pest Manag. Sci.*, *75*, 658-666.

Morandin, L.A., Long, R.F., & Kremen, C. (2014). Hedgerows enhance beneficial insects on adjacent tomato fields in an intensive agricultural landscape. *Agric. Ecosyst. Environ.*, *189*, 164-170.

Morandin, L. A., Long, R. F., & Kremen, C. (2016). Pest control and pollination cost–benefit analysis of hedgerow restoration in a simplified agricultural landscape. *J. Econ. Entomol.*, *109*, 1020-1027.

Phillips, B. W., & Gardiner, M. M. (2015). Use of video surveillance to measure the influences of habitat management and landscape composition on pollinator visitation and pollen deposition in pumpkin (*Cucurbita pepo*) agroecosystems. *PeerJ*, *3*, e1342.

Phillips, B. W., & Gardiner, M. M. (2016). Does local habitat management or large-scale landscape composition alter the biocontrol services provided to pumpkin agroecosystems?. *Biol. Control*, *92*, 181-194.

Pfister, S. C., Eckerter, P. W., Schirmel, J., Cresswell, J. E., & Entling, M. H. (2017). Sensitivity of commercial pumpkin yield to potential decline among different groups of pollinating bees. *R. Soc. Open Sci.*, *4*(5), 170102.

Rundlöf, M., Lundin, O., & Bommarco, R. (2018). Annual flower strips support pollinators and potentially enhance red clover seed yield. *Ecol. Evol.*, *8*, 7974-7985.

Sardiñas, H. S., & Kremen, C. (2015). Pollination services from field-scale agricultural diversification may be context-dependent. *Agric. Ecosyst. Environ.*, *207*, 17-25.

Scheid, B. E., Thies, C., & Tscharntke, T. (2011). Enhancing rape pollen beetle parasitism within sown flower fields along a landscape complexity gradient. *Agric. Forest Entomol.*, *13*, 173-179.

Sutter, L., Albrecht, M., & Jeanneret, P. (2018). Landscape greening and local creation of wildflower strips and hedgerows promote multiple ecosystem services. *J. Appl. Ecol.*, *55*, 612-620.

Thies, C., & Tscharntke, T. (1999). Landscape structure and biological control in agroecosystems. *Science*, *285*(5429), 893-895.

Tschumi, M., Albrecht, M., Entling, M. H., & Jacot, K. (2015). High effectiveness of tailored flower strips in reducing pests and crop plant damage. *Proc. R. Soc. B*, *282*, 20151369.

Tschumi, M., Albrecht, M., Collatz, J., Dubsky, V., Entling, M. H., Najar‐Rodriguez, A. J., & Jacot, K. (2016a). Tailored flower strips promote natural enemy biodiversity and pest control in potato crops. *J. Appl. Ecol.*, *53*, 1169-1176.

Tschumi, M., Albrecht, M., Bärtschi, C., Collatz, J., Entling, M. H., & Jacot, K. (2016b). Perennial, species-rich wildflower strips enhance pest control and crop yield. *Agric. Ecosyst. Environ.*, *220*, 97-103.

Venturini, E.M., Drummond, F.A., Hoshide, A.K., Dibble, A.C., & Stack, L.B. (2017). Pollination reservoirs in lowbush blueberry (Ericales: Ericaceae). *J. Econ. Entomol.*, *110*, 333-346.

Woltz, MJ, R Isaacs, DA Landis. 2012. Landscape structure and habitat management differentially influence insect natural enemies in an agricultural landscape. *Agric. Ecosyst. Environ.*, 152, 40-49.
