# Supplementary material for: The effectiveness of flower strips and hedgerows on pest control, pollination services and crop yield: a quantitative synthesis
Source: Ecol Lett. 2020 Aug 18;23(10):1488–98. doi: 10.1111/ele.13576 (PMC7540530; doi:10.1111/ele.13576)
Supplement: Supplementary file 4 — Table S3 [file ELE-23-1488-s006.docx]

*Supporting information* to Albrecht *et al.*: **Global synthesis of the effectiveness of flower strips and hedgerows on pest control, pollination services and crop yield**

**Supporting Table S3.** Summary of results of statistical analysis testing for hump-shaped relationship of the proportion of semi-natural habitat at the landscape scale (1 km radius) and the interactions with local flower planting (crop with or without adjacent planting).
