# Supplementary material for: The effectiveness of flower strips and hedgerows on pest control, pollination services and crop yield: a quantitative synthesis
Source: Ecol Lett. 2020 Aug 18;23(10):1488–98. doi: 10.1111/ele.13576 (PMC7540530; doi:10.1111/ele.13576)
Supplement: Supplementary file 5 — Table S4 [file ELE-23-1488-s002.docx]

*Supporting information* to Albrecht *et al.*: **Global synthesis of the effectiveness of flower strips and hedgerows on pest control, pollination services and crop yield**

**Supporting Table S4.** Summary of results of statistical analyses of effects of flower strips on crop yield. In addition to the effect of flower strip (flower strip present or not) results of statistical analyses testing for effects of potential drivers of the effectiveness flower strips on crop yield (within-field distance from flower strip, time since establishment and landscape simplification) are shown.
