# Supplementary material for: The effectiveness of flower strips and hedgerows on pest control, pollination services and crop yield: a quantitative synthesis
Source: Ecol Lett. 2020 Aug 18;23(10):1488–98. doi: 10.1111/ele.13576 (PMC7540530; doi:10.1111/ele.13576)
Supplement: Supplementary file 6 — Table S5 [file ELE-23-1488-s001.docx]

*Supporting information* to Albrecht *et al.*: **The effectiveness of flower strips and hedgerows on pest control, pollination services and crop yield: a quantitative synthesis**

**Supporting Table S5.** Summary of linear mixed effect model analysis exploring (1) whether the effect of flower plantings (flower strips and hedgerows) on crop pollination service delivery varied with the degree of dependency of the crop on insect pollination (interaction floral planting × insect dependency); and (2) whether in studies reporting crop yield, the effect of flower strips on yield varied with the degree of insect pollinator dependency of the crop (interaction flower strip × insect dependency). Study was included as a random intercept to account for the hierarchical structure of the data with field measures nested within study. As different studies used different methods and measures to quantify pollination services and yield, data was standardized using *z*-scores prior to analysis (see Materials and Methods section of the main text). Degree of insect pollinator dependency was quantified according to guidelines by Galllai & Vaissière (2009), based on data from Klein *et al.* (2007).

**References**

Gallai, N. & Vaissière, B. (2009). Guidelines for the economic valuation of pollination services at a national scale. Guidelines for the economic valuation of pollination services at a national scale. FAO, Rome, Italy.

Klein, A.M., Vaissiere, B.E., Cane, J.H., Steffan-Dewenter, I., Cunningham, S.A., Kremen, C. & Tscharntke, T. (2007). Importance of pollinators in changing landscapes for world crops. P*roc. Royal Soc. B*, 274, 303–313.
